# Nanomaterials and Essential Oils as Candidates for Developing Novel Treatment Options for Bovine Mastitis

**DOI:** 10.3390/ani11061625

**Published:** 2021-05-31

**Authors:** Andra Sabina Neculai-Valeanu, Adina Mirela Ariton, Bianca Maria Mădescu, Cristina Mihaela Rîmbu, Şteofil Creangă

**Affiliations:** 1 Research and Development Station for Cattle Breeding Dancu, Sos. Iasi-Ungheni no. 9, 707252 Dancu, Romania; mirela.ariton@scdb-dancu.ro (A.M.A.); biancamadescu@uaiasi.ro (B.M.M.); 2 Department of Fundamental Sciences in Animal Husbandry, Faculty of Food and Animal Sciences, Iasi University of Life Sciences (IULS), Mihail Sadoveanu Alley no. 8, 700490 Iasi, Romania; screanga@uaiasi.ro; 3 Department of Public Health, Faculty of Veterinary Medicine, Iasi University of Life Sciences (IULS), Mihail Sadoveanu Alley no. 8, 700490 Iasi, Romania; crimbu@uaiasi.ro

**Keywords:** antimicrobial resistance, cattle, essential oils, graphene, mastitis, nanoparticles

## Abstract

**Simple Summary:**

Bovine mastitis is a highly prevalent and expensive illness that leads to enormous financial losses to dairy industries. Although significant progress has been made in the control and therapy of mastitis, the frequency of this disease continues to be high in livestock herds, having a negative influence on productive parameters and implicitly on economic indices in dairy farms. The opportunity to address this problem resides in the fact that a countries’ share of the community market will only be maintained by increasing milk quality. It is imperative for cattle farmers, milk producers and national economies to obtain milk according to EU standards, in order for it to be valued at advantageous, competitive prices. This objective may be achieved by keeping the incidence of mastitis under very strict control, using novel treatment options.

**Abstract:**

Nanomaterials have been used for diagnosis and therapy in the human medical field, while their application in veterinary medicine and animal production is still relatively new. Nanotechnology, however, is a rapidly growing field, offering the possibility of manufacturing new materials at the nanoscale level, with the formidable potential to revolutionize the agri-food sector by offering novel treatment options for prevalent and expensive illnesses such as bovine mastitis. Since current treatments are becoming progressively more ineffective in resistant bacteria, the development of innovative products based on both nanotechnology and phytotherapy may directly address a major global problem, antimicrobial resistance, while providing a sustainable animal health solution that supports the production of safe and high-quality food products. This review summarizes the challenges encountered presently in the treatment of bovine mastitis, emphasizing the possibility of using new-generation nanomaterials (e.g., biological synthesized nanoparticles and graphene) and essential oils, as candidates for developing novel treatment options for bovine mastitis.

## 1. Introduction

The livestock industry is presently centered on the genetic improvement of dairy cattle, with the increase in yield milk being nearly double compared to 1960 [1]. The dairy sector accounts today for around 15% of the total agricultural output. However, selecting for a higher yield of milk production is correlated with the appearance of certain problems in the udders of dairy cows, such as higher percentage of somatic cells and a higher incidence of mastitis [2].

Bovine mastitis is the most prevalent and expensive illness affecting milk herds worldwide. The disease leads to enormous financial losses to dairy industries as a result of decreased milk production and quality, condemnation of milk due to antibiotic residues and culling of chronically infected cows, as well as associated therapy costs. When zoonotic pathogens are involved, mastitis may pose a serious health risk due to the dispersion of bacteria and toxins in milk.

In the clinical form, symptoms may vary depending on the causative agent, while in the subclinical form, there are no visible changes in the aspect of the udder or milk. Monitoring the number of somatic cells is a widely used practice in the European Union (EU) for assessing milk quality [3,4].

Although significant progress has been made in the control and therapy of mastitis, the frequency of this disease continues to be high in some livestock herds, having a negative influence on productive parameters and implicitly on economic indices in dairy farms. In Europe, the economic losses caused by mastitis to the dairy industry have been estimated at around 2 billion euros annually. A poll conducted in the major milk-producing countries indicates that this disease affects, each year, between 15% and 20% of the dairy cow population. Based on the origin of the pathogen, mastitis may be classified as environmental, caused by pathogens from the environment and contagious, spread from other infected quarters [5]. According to previously conducted studies, the most common bacteria causing intramammary inflammation are *Staphylococcus aureus*, *Streptococcus agalactiae*, *Escherichia coli* and *Streptococcus uberis* [6,7,8].

The opportunity to address this problem resides in the fact that a country’s share of the community market will only be maintained by increasing milk quality. It is imperative for cattle farmers, milk producers, and national economies to obtain milk according to EU standards in order to value it at advantageous, competitive prices. This objective may be achieved by keeping the incidence of bovine mastitis under very strict control using novel treatment options [9].

By the end of 2027, the worldwide bovine mastitis market is expected to hit USD 1.84 billion. It is estimated that recent breakthroughs, such as nanomaterials-based solutions for the treatment and control of bovine mastitis, will lead the way to an interesting future of the global market. According to the report entitled “Bovine Mastitis Market Size, Share & COVID-19 Impact analysis, by type (Clinical, and Sub-Clinical), by product (antibiotics, and others), by route of administration (Intra-mammary, and Systemic), by therapy (Lactating Period and Dry Period) and Regional Forecast, 2020–2027”, published by Fortune Business Insights, the market, which was estimated in 2019 at USD 1.23 billion, is forecasted to experience a compound annual growth rate (CAGR) of 5.2% during 2020–2027 [10]. The growth of the European market will be powered by a growing number of business alliances aimed at developing novel treatment options for bovine mastitis in a number of countries, including France, Germany and the UK.

Nanomaterials have been used for diagnosis and therapy in the human medical field, while their application in veterinary medicine, for drug delivery, diagnostic and cell sorting or antimicrobials [11,12,13,14] and animal production, especially animal nutrition [15,16,17,18], is still relatively new. Nanotechnology, however, is a rapidly growing field, offering the possibility of manufacturing new materials at the nanoscale level, with the formidable potential to revolutionize the agri-food sector by offering novel treatment options for prevalent and expensive illnesses such as bovine mastitis.

This review summarizes the challenges encountered presently in the treatment of bovine mastitis, emphasizing the possibility of using new-generation nanomaterials, such as biological synthesized nanoparticles and graphene, and essential oils as candidates for developing novel treatment options for bovine mastitis.

## 2. Farm Model with “Low Antibiotic Consumption”

Owing to inadequate monitoring and data collection in many nations, figures of overall annual global antibiotic use in the EU’s agricultural sector vary greatly, from 1770.4 tonnes per year in Spain to 0.6. tones in Iceland [19]. The differences between countries are explained by a variety of factors. To begin with, animal populations, as well as production systems, differ considerably from one country to other. A second aspect is the dose and treatment period, which are not included in the results because there are significant differences across countries regarding the pharmaceutical formulations used and the length of the therapy. Different antibiotics, as well as different amounts, are needed depending on whether pigs, cows, sheep or chickens are treated. For this reason, one of the key points of the ESVAC: Vision, Strategy and Objectives for 2016–2020 was the development of a system for collecting harmonized and standardized data on antimicrobial use by animal species in order to conduct a more systematic study of patterns in antimicrobial use [20].

**Figure 1:**
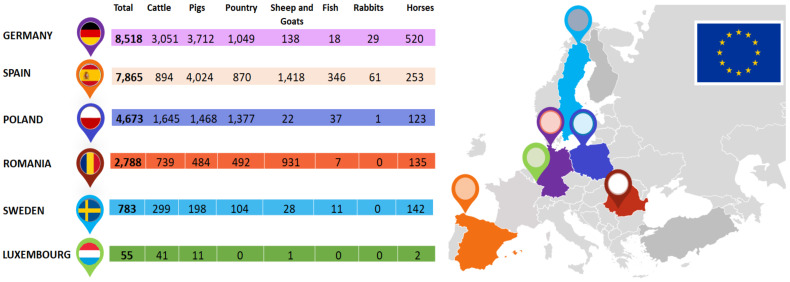
Estimated PCU (in 1000 tonnes) of the population of food-producing species (including horses) for 2018 in some EU member states according to the European database of sales of veterinary antimicrobial agents.

In 2017, the European Commission and the EMA held a workshop on the compilation of data on veterinary antimicrobial use in EU countries. Stakeholders were mostly in agreement on the importance of collecting data on antimicrobial use by animal species, as well as the advantages of doing so; therefore, on February 6th 2018, ESVAC adopted a guide regarding the collection and provision of national data on antimicrobial use by animal species/categories. Since the volume of veterinary antimicrobial agents sold across the EU member states is related to the animal population structure, in order to normalize the sales statistics for the animal population that may be treated with antimicrobial agents, the European Commission requested that the European Centre for Disease Prevention and Control (ECDC), European Food Safety Authority (EFSA) and European Medicines Agency (EMA) use a common scientific opinion regarding some indicators that may be used for the surveillance of antimicrobial resistance and antimicrobial consumption in humans and food-producing animals [21]. The European Surveillance of Veterinary Antimicrobial Consumption (ESVAC) employed a population correction unit (PCU) as a proxy for the size of the animal population [19] (Figure 1).

The proposed indicators used presently for antimicrobial surveillance are classified as primary indicators—overall sales of veterinary antimicrobials expressed in mg/PC and secondary indicators—sales of 3rd- and 4th-generation cephalosporins in mg/PCU; sales of quinolones, specifying the % of fluoroquinolones in mg/PCU, and sales of polymyxins in mg/PCU [21]. This format is considered more suitable for comparing sales data across countries and years because it accounts for variations in the size and composition of the animal population in the EU member states [19,20].

Veterinarians, fishermen, other livestock industry players, EU Member States, the European Commission and the European Medicines Agency have all worked together to reduce sales of antimicrobials. National strategies for antibiotic stewardship in livestock, limitations regarding the use of certain antimicrobials in food-producing animals, the removal of antibiotics as growth promoters and EU good practices guidelines are among the measures taken to decrease veterinary antimicrobial purchases across Europe. These measures are part of the EU’s One Health Action Plan to Tackle Antimicrobial Resistance (AMR). According to The European Medicines Agency (EMA), between 2011 and 2017, the total sales of veterinary antibiotics in Europe fell by more than 32% [22].

**Figure 2:**
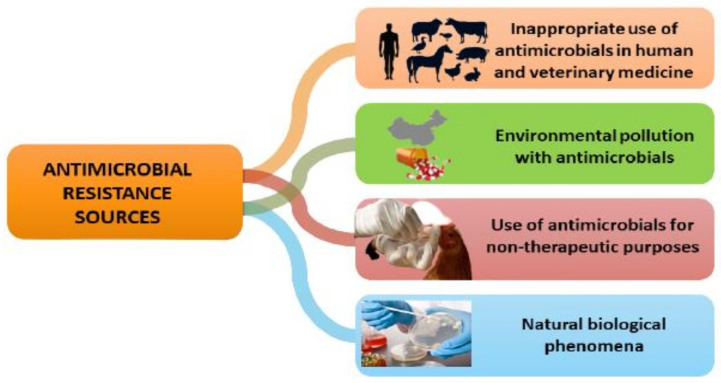
Main sources of antimicrobial resistance.

Antimicrobial resistance is a worldwide issue that affects all countries and populations, regardless of wealth or socioeconomic status, but the challenges are proportionally higher in underdeveloped countries and emergent economies [23]. Resistant organisms have no territorial or biological boundaries: they may quickly propagate by the migration of humans, plants, food or water, and certain resistance genes can be transferred from one species to another [9]. Apart from being a naturally occurring phenomenon, antimicrobial resistance occurs in both humans and animals when antibiotics are used excessively or inappropriately, subsequently leading to environmental pollution as well (Figure 2).

Studies conducted by various authors showed that sub-lethal antibiotic therapy may lead to multidrug resistance due to reactive oxygen species (ROS)-induced mutagenesis [24,25,26,27]. Moreover, a study conducted by Li et al. (2021) [28] in a simulated sublethal concentration of copper and tetracycline co-contaminated environment showed that the contamination of the environment with metals and antibiotics may accelerate the emergence of antibiotic-resistant bacteria and their dissemination.

**Figure 3:**
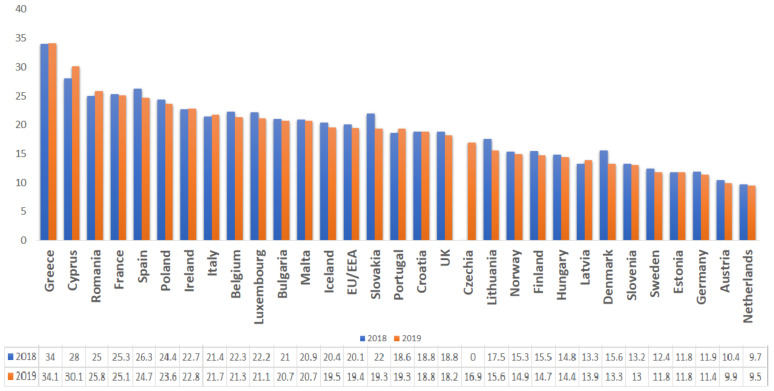
Total consumption (community and hospital sector) of antibacterials for systemic use (ATC group J01) by country, EU/EEA, 2018–2019 (expressed as DDD per 1000 inhabitants per day).

**Figure 4:**
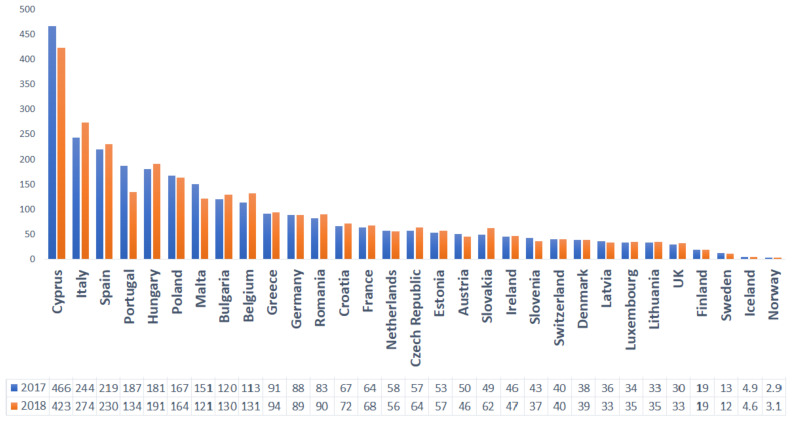
Overall sales for food-producing animals (including horses), in mg per population correction unit (mg/PCU), of the various veterinary antimicrobial classes, by country, for 2017 and 2018.

Given the near interdependence between humans and animals that share the same ecosystem, excessive veterinary use of these compounds poses a potential public health threat [29,30]. For example, in Romania, the high consumption rates and the widespread use of broad-spectrum antibiotics for both human and animals has led to growing concerns over antimicrobial resistance. The country ranked 3rd among the EU countries for total consumption (community and hospital sector) of antibacterials for systemic use (ATC group J01) in 2019 [31] (Figure 3) and 12th regarding the use of antimicrobials for food-producing species (including horses), expressed in mg per population correction unit (mg/PCU), in 2018 (Figure 4) [22]

The situation raised the awareness of the European Public Health Alliance (EPHA), which subsequently commissioned a study regarding the current AMR situation in Romania. According to the conducted study, Romania is “in the red zone” due to inadequate multidisciplinary collaboration between clinicians, microbiologists and epidemiologists; the limited collaboration between human and veterinary domains with regard to AMR; and the lack of robust data regarding the infections with “superbugs” in both humans and animals. Unfortunately, several other EU member states are confronted with similar threats, which tend to affect Europe’s safety network [32], thus becoming a growing cross-border threat to public health in Europe and around the world [33,34]. Antimicrobial-resistant infections are believed to kill 700,000 people per year around the world. If urgent actions are not taken, the number is expected to climb to 10 million people by 2050, with associated costs of $100 trillion. Many of the Sustainable Development Goals (SDGs) are also compromised by AMR. The World Bank predicts that an additional 28 million people will be plunged into extreme poverty by 2050 if AMR is not regulated adequately [35,36,37,38,39].

One of the specific objectives of the new Commune Agricultural Policy (CAP), after 2020, is to improve the response of EU agriculture to society’s food and health demands. The European Commission’s proposal for a new CAP contains several key points to support the transition towards a European farm model with “low antibiotic consumption”. If successful, this will help to achieve the EU’s vision of a region of “best practices” to counteract AMR, as stipulated in the Health Action Plan against AMR. The new policies represent an opportunity for the transition to high-health production systems that will provide healthy, more profitable livestock, improved financial results for farmers, and decreased antibiotic use [40,41,42,43,44].

**Figure 5:**
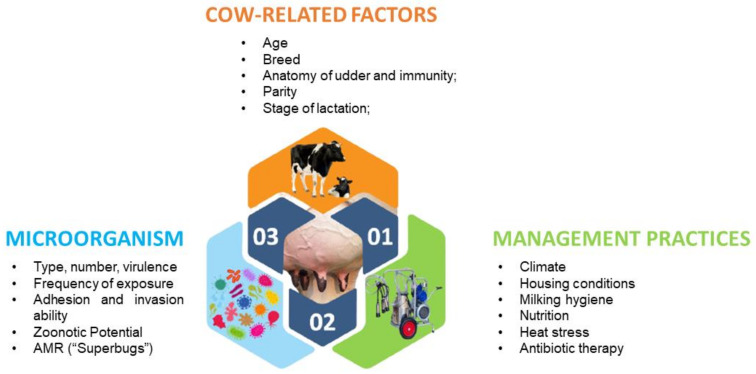
Host, microorganism and environmental factors with the potential of inducing mastitis in dairy cows.

Bovine mastitis is the most costly disease affecting cows worldwide. Reduced milk production, changes in milk composition, discarded milk, higher replacement costs, additional labour, care costs and veterinarian services all add to substantial economic losses for dairy farmers and the milk processing sector. The risk factors that predisposes dairy cows to mastitis may be classified as cow-related, microorganism-related or management-practices-related risk factors. Some of the cow-related risk factors are age, lactation, somatic cell count, breed, udder and teat anatomy and the immune status of the animal (Figure 5) [45,46,47,48,49,50,51,52].

The main pathogens that cause mastitis include infectious microorganisms that survive and proliferate on skin and teat wounds, such as *Streptococcus agalactiae*, *Staphylococcus aureus* and *Streptococcus dysgalactiae*, as well as environmental microorganisms such as *Streptococcus uberis*, *Escherichia coli* and other coliforms [53,54]. In comparison to contagious pathogens, environmental pathogens typically do not survive on the skin of the cow’s udder and teat; they are better characterized as pathogens that are opportunistic, often reside in the bedding and housing, searching for the ability to cause an infection. When the natural immunity of cows is affected, bacteria enter the cow’s mammary glands through the teat canal, where they colonize, proliferate, and release toxins, damaging the mammary gland cells. The most common Gram-positive pathogen believed to be involved with different types of clinical and sub-clinical mastitis is *Staphylococcus aureus*. The main reservoir for *Staphylococcus aureus* is the cows with chronic infection in the mammary gland; thus, improved udder hygiene will shield healthy cows from infected ones, thereby reducing infection. The intra-mammary administration of antibiotics such as penicillin, ampicillin, tetracycline and gentamycin has always been the first choice for the therapy against bovine mastitis. However, the inappropriate use and overuse of antibiotics in many bacterial species have resulted in an alarming increase in multi-drug-resistant bacterial diseases [55,56]. Since some strains are resistant to almost all frequently available agents such as beta-lactams, tetracyclines and amino-glycosidases, there is a major worldwide threat to public health. Since current treatments are becoming progressively more ineffective in resistant bacteria, there is a growing need to increase funding for research and development of new products and technologies to tackle AMR in humans and animals. Thus, the development of innovative products based on both nanotechnology and phytotherapy may directly address a major global problem, antimicrobial resistance, while providing a sustainable animal health solution that supports the production of safe and high-quality food products.

## 3. New Approaches for the Development of Bovine Mastitis Products—Nanomaterials and Essential Oils

Not surprisingly, many recent research studies have been aimed at identifying possible alternatives to antibiotic therapy in order to prevent antibiotic resistance [57,58,59,60,61,62,63,64,65,66,67,68,69,70,71,72]. A study by Bouari et al. (2016) [73] assessed the in vitro antimicrobial susceptibility of bacteria isolated from mastitic milk to design specific control programs for bovine mastitis in the Transylvanian area (Romania). Among the pathogens identified was *Staphylococcus aureus*, in which the authors observed increased resistance to penicillin and tetracycline.

Since ancient times, people have widely used natural herbal products as medicines against various diseases. Almost 25% of the main pharmaceutical compounds and their derivatives available today are produced from natural resources [74], so phytotherapy may be a good starting point for the development of antimicrobial products.

The antimicrobial, immunomodulatory efficacy of medicinal plants has been highlighted in numerous studies over the last few years [75,76,77,78], while the potential applications of natural polymers such as chitosan and eco-friendly metal nanoparticles for the development of innovative antimicrobial products have been highlighted in studies by Burdusel et al. (2018) [79], Baican and Vasile (2018) [80], Olaru et al. (2019) [81], Ahmed et al. (2020) [82] and Sánchez-López et al. (2020) [83].

### 3.1. Essential Oils and Vegetal Extracts

Essential oils (EO), which may be derived from various parts of a plant, contain compounds of various types that have both physiological and therapeutic benefits. They can act either individually or in synergy, with antibacterial, antifungal, antiviral and anti-inflammatory properties. For example, the biological activities of cinnamon, including its anti-inflammatory characteristics, which are determined by their phenolic and volatile compounds, have been the central subject of several studies conducted in recent years. Studies in vitro and in vivo showed that cinnamon essential oil has bactericidal effects against bovine mastitis pathogenic isolates by impairing the membrane integrity of bacteria, thus constituting an alternative organic antimicrobial to ensure milk safety [84]. Furthermore, cinnamon may reduce both inflammation and the damage of the mammary tissue associated with bovine mastitis disease [85,86].

A study conducted by Nardoni et al. (2018) [87] showed the antialgal efficacy of 30 types of essential oils against *Prototheca zopfii* and *Prototheca blaschkeae*, agents that determine protothecal mastitis, an emergent animal health problem in dairy herds. Furthermore, the antimicrobial activity of other essential oils such as thyme, oregano or lavender on common bovine mastitis pathogen such as Staphylococcus sp., Streptococcus sp., Bacillus cereus, Escherichia coli as well as the effect of other plant extract was investigated in different studies [78,88,89,90,91] (Table 1).

Worldwide products based on herbal extracts and essential oils under the form of a spray or ointment have been used for the treatment of mastitis in ruminants, especially in organic farms [103]. However, despite their great potential as non-antibiotic antibacterial agents, essential oils have several disadvantages, including instability, intense smell, biodegradability and low solubility in certain solutions. These issues have limited the applications of essential oils in the food and medical industries. Moreover, newer research has shown that the effectiveness of phytotherapy could be greatly improved, including against antibiotic-resistant pathogens, by its association with metal nanoparticles, enabling the development of systems for the distribution and controlled release of essential oils [104,105].

### 3.2. Metallic Nanoparticles

Metallic nanoparticles are submicron-size entities with a metal core composed of inorganic metal or metal oxide, and they are usually covered by a shell layer of organic or inorganic material or metal oxide. The use of nanoparticles in numerous fields such as energy [106,107], biomedicine [108,109,110,111,112], assisted reproduction [113,114,115], wood industry [116,117,118], food industry [119,120,121] and agriculture [122,123,124,125,126,127] has risen exponentially in recent years.

Metallic nanoparticles may be synthesized by various methods, through top-down (e.g., laser ablation, ball milling and chemical etching) and bottom-up approaches (e.g., chemical vapor deposition, sol-gel process, spray pyrolysis and green synthesis). The top-down strategy implies that the bulk material is converted into small, nano-sized structures, using different reagents and physical treatments. In the bottom-up approach, nanoparticles are grown to a specific size and shape from simpler molecules [128,129,130,131,132].

**Figure 6:**
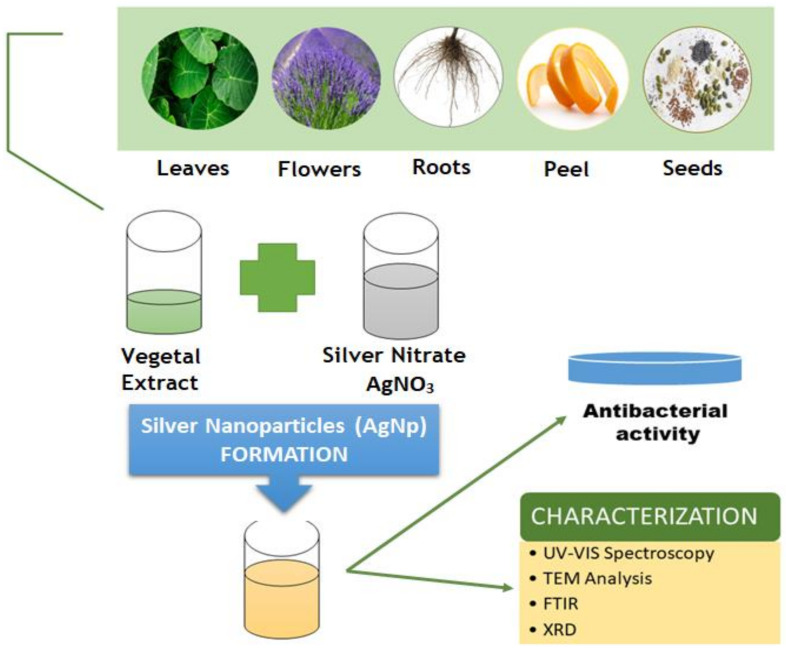
Schematic representation of silver nanoparticles green synthesis.

In contrast with the nanoparticles derived from physical and chemical synthesis, which pose a high health and toxicity risk, the green synthesized nanoparticles are safer, eco-friendly alternatives because the obtaining process is clean and non-toxic [133,134,135]. Green synthesized metallic nanoparticles may be obtained using leaves [136,137,138], flowers [139,140], seeds [141,142], peel [143,144] or roots [145,146] (Figure 6). Moreover, the production of different types of metallic nanoparticles using agro-waste has become more and more popular [147,148,149,150].

The antimicrobial effects of metallic nanoparticles (NPs) are mainly due to the release of metallic ions, disruption of the cell membrane/wall, generation of ROS and inhibition of proper DNA replication [151,152,153,154]. The reaction parameters such as pH, temperature and reaction time may be adapted in order to produce metallic nanoparticles with desired shape and size [155,156].

These physical characteristics may be further exploited for the development of antimicrobial products. Besides their antibacterial activity, metallic nanoparticles may be effective against bovine mastitis pathogens (Table 2) and methicillin-resistant bacteria, inhibiting biofilms [157,158,159].

Moreover, in the treatment of bovine mastitis, nanoparticles show greater effects on bacteria than their micro-counterparts and may serve as potential delivery systems because they may be ingested by phagocytes [11,167]. According to Pinheiro Machado et al. (2019) [168], nanoparticles may be used as a carrier for the efficient delivery of nano-propolis formulation [169,170,171,172]. Due to its improved antimicrobial activity as well as low cytotoxicity, this type of therapeutical alternative for bovine mastitis control is gaining popularity in organic dairy farms. Furthermore, studies conducted by Soni and Yadav (2016) [173], Krishna et al. (2017) [174], Mohsenabadi et al. (2018) [175] and Vasile et al. (2020) [176], have shown that nanogels may be an outstanding method to address the therapeutic challenges posed by intracellular pathogens such as *Staphylococcus aureus*.

More recently, the potential of new-generation antibacterial materials such as graphene-based nanomaterials were also exploited [177,178]. Graphene oxide (GO) and reduced graphene oxide (rGO) showed excellent potential in drug delivery and photodynamic/photothermal therapy, as well as antimicrobial properties due to their composition and physicochemical characteristics [178,179,180]. According to some studies, these materials may be used in synergism with other biocidal material, such as rare metal nanoparticles [181,182] or essential oils [183] with applications in various sectors such as biomedicine and food safety. By enriching their antibacterial properties, these materials may be a promising tool even against multidrug-resistant agents such as Methicillin-resistant *Staphylococcus aureus* (MRSA) [184,185,186,187].

According to recent studies, graphene-based materials are being used successfully in tissue engineering and regenerative medicine due to their potential to improve both the wound-healing process and infection control at the injury site [188,189,190,191]. Since the lesions on teat skin commonly harbor bacteria that may cause mastitis, consequently leading to partial or complete damage to udder tissues and reduced milk production, the anti-inflammatory and regenerative properties of graphene may be exploited in the development of innovative formulations. According to Pelin et al. (2017) [192], the cytotoxicity of graphene oxide at skin level is low, which is why graphene-based products may be safely used for the development of a nanogel with topical applications on the udder skin for the treatment and prevention of bovine mastitis.

## 4. Challenges

The administration of oral pharmacological substances is, so far, the most convenient and accepted way of administering drugs, but in recent years, the science of drug delivery has reached unprecedented landmarks, thus, presently there is considerable interest in the administration of drugs to the systemic circulation, in a non-invasive manner, through mucosal routes (ophthalmic, rectal, vaginal) or skin [193]. The development of innovative drug delivery methods and the nanotechnological advancements have catapulted topical drug delivery to a whole new era [194,195]. However, delivering the active substances into the bloodstream topically, using a non-invasive manner, remains a challenge.

An effective topical formulation must meet two concomitant characteristics, namely being thermodynamically stable while allowing the transport of the active substance through the stratum corneum of the skin, thus guaranteeing that the necessary therapeutic amount of drug reaches the targeted region [196,197,198,199,200]. A poor percutaneous penetration capability for most topical antibacterial and anti-inflammatory formulations is one of the main causes compromising their therapeutic effects [201]. The transdermal delivery of active compounds is a challenge that may be solved by hydrogels [202,203,204]. These promising 3D materials have demonstrated increasing applications in the encapsulation and topical delivery of drugs due to specific properties such as high hydrophilicity, unique three-dimensional network, fine biocompatibility and cell adhesion [204,205]. These distinctive properties give hydrogels the ability to protect the pharmacological substance against biodegradation while also ensuring its controlled and regulated release. Furthermore, hydrogels may be functionalized using different nanomaterials such as nanotubes, graphene, dendrimers, ceramic and metallic nanoparticles. The integration of nanomaterials within a polymeric hydrogel network is an appealing method for personalizing the mechanical properties of the hydrogels and/or improving the responsiveness to external stimuli [206].

Nanoparticles may act as a multifunctional cross-linking point in three-dimensional polymeric chains; thus, the size and shape of the nanoparticles may influence the mechanical strength of the nanocomposite hydrogel [207,208]. Metallic nanoparticles are well known for their antimicrobial activity against a broad range of bacteria strains, including an antibiotic-resistant strain, making them suitable for the formulation of nanogels with antimicrobial activity [201,207,209,210,211,212]. 

Graphene-based nanomaterials also show impressive antimicrobial characteristics [213,214]. However, due to their powerful inter-plane interactions, they tend to aggregate, which is why their surface area and mode of action is presently limited. Therefore, different functionalization and surface modification with metal ions/oxides/sulfides NPs, polymers and enzymes must be conducted on graphene in order to decrease aggregation and improve biosafety.

Many attempts have been made over the last several years to produce selective drug delivery systems that allow medication to be delivered to particular locations such as muscles, tissues and cells in the body to enhance treatment management. Nanogels are among the most promising drug delivery systems due to their unique structure that combines the properties and features of both hydrogels and nanoparticles.

## 5. Conclusions

Routine animal health assessments, as a core technique for infection prevention and control on farms, as well as the dissemination of best practices at the regional and national levels, may enhance animal health and food safety. This strategy might help limit food contamination and antibiotic consumption on farms. In the same way as reducing human infection reduces antimicrobial use (and so the risk of AMR), reducing animal infection reduces antimicrobial use and hence the risk of AMR.

However, more resistance might naturally arise as a result of evolution. Therefore, any strategy to counteract AMR must have an emphasis on enhancing the manufacturing and use of highly effective new antimicrobials, vaccines and infection prevention therapies.

Nanomaterials have great potential in the development of new drug formulas, as an alternative to current therapy products, the benefits of these new types of formulas being determined both by the diversity of excipients and by their particularly advantageous specific properties such as relative abundance, biocompatibility, biodegradability, non-irritability, innocuity and low-cost price.

In addition, essential oils are recognized as biocompatible, biodegradable and non-toxic, and due to their ability to penetrate the skin barrier, these compounds may be used alongside other nanomaterials to improve both the transdermal transport of the newly developed products, as well as their efficiency.

## Figures and Tables

**Table 1:** Summary of recent studies regarding the use of essential oils (EO) and plant extracts against mastitis pathogens.

Type of Oil/Plant Extract	Type of Study	Tested Pathogens	Effect	References
Siam weed EO (*Chromolaena squalida*) Guabiroba Verde EO (*Campomanesia sessiliflora*) Rapanea punctata EO (*Myrsine guianensis*) (*Matayba guianensis*) EO Negramina EO (*Siparuna guianensis*) Canelinha EO (*Ocotea minarum*) Endlicheria EO (*Endlicheria paniculata*)	In vitro	*Staphylococcus aureusEscherichia coli Escherichia coli* (β−lactamase producer) *Pseudomonas aeruginosa*	All of the tested oils demonstrated moderate to excellent activity against four bacterial species, including *Salmonella Typhi* and oxacillin-resistant *Staphylococcus aureus*.	de Jesus et al. 2020 [92]
FMexican Avocado Seed (*Persea americana var. drymifolia*)	In vitro	*Staphylococcus aureus*	Lipid extract from avocado seed inhibits the *Staphylococcus aureus* internalization into bovine mammary epithelial cells (bMECs) and modulates the innate immune response (IIR)	Báez-Magaña et al. 2019 [93]
Pink Powderpuff (*Calliandra surinamensis*)	In vitro	*Staphylococcus* isolates from either bovine (Ssp6PD and Sa) or caprine (Ssp5D and Ssp01) mastitic milk samples	*Calliandra surinamensis* leaf pinnulae lectin displayed a bacteriostatic and antibiofilm agent against certain bovine and caprine mastitis isolates. When used in conjunction with either ampicillin (against one isolate) or tetracycline (against two isolates), it showed synergistic effect.	Procópio et al. 2019 [94]
Southern blue gum (*Eucalyptus globulus*) Walnut (*Juglans regia*)	In vitro	*Staphylococcus aureus*	*Eucalyptus globulus* extract alone appeared to have a bacteriostatic effect against *Staphylococcus aureus*, up to 8 hours of incubation. When opposed to the positive control, *Eucalyptus globulus* and *Juglans regia* extracts alone had a minor inhibitory effect over time.	Gomes et al. 2019 [95]
Black Myrobalan (*Terminalia chebula*) extract	In Vitro	*Staphylococcus aureus*	The 500 µg/mL concentration of *Terminalia chebula* ethyl acetate extract was as effective as standard amoxicillin	Kher et al. 2019 [96]
Rose Myrtle Rhodomyrtus tomentosa (*Rose myrtle*) leaves	In vitro In vivo	*Staphylococcus aureus*	The ethanolic extract showed good antibacterial activity in vitro, a reduction of activity being observed in vivo.	Mordmuang et al. 2019 [97]
Wild cabbage (*Brassica oleracea*)	In vitro	*Staphylococcus aureus Escherichia coli Klebsiella pneumoniae*	Interferes in the mechanisms of action of genes such as MTOR and TP53, thus may be a possible alternative for developing herbal formulations for bovine mastitis.	Sobrinho Santos et al. 2019 [98]
Piperina EO (*Minthostachys verticillata*)	In vitro	*Escherichia coli Bacillus pumilus Enterococcus faecium*	EO affected the formation of biofilm and revealed the antibacterial capacity of EO and limonene.	Cerioli et al. 2018 [90]
Oregano EO (*Origanum floribundu*) Morrocan Thyme EO (*Thymus ciliatus*) Rosemary EO (*Rosmarinus officinalis*)	In vitro	*Candida albicans*	The three essential oils showed highly anticandidal activity, with values ranging from 15.02 to 31.08 g/mL.	Ksouri et al. 2017 [99]
Cinnamon EO (*Cinnamomum zeylandicum*) Geranium EO (*Pelargonium graveolens*) Clove EO (*Syzygium aromaticum*) Thyme EO (*Thymus vulgaris*) Lavender EO (*Lavandula angustifolia*) Basil EO (*Ocimum basilicum*) Rosemary EO (*Rosmarinus officinalis*) Clary sage EO (*Salvia sclarea*)	In vitro	Eight strains of *Prototheca zopfii* isolated from mastitic milk	Many of the oils tested were effective against algal strains, but cinnamon, clove, and thyme were the most effective.	Grzesiak et al. 2016 [91]
Oregano EO (*Origanum vulgare*)	In vivo	*Staphylococcus aureus* and *Escherichia coli*	In the group of cows treated intramammary with oregano essential oil (OEO), the number of somatic cells (SCCs) and number of white blood cells (WBC) were significantly decreased and *Staphylococcus aureus* and *Escherichia coli* were not present in milk samples.	Cho et al. 2015 [100]
Thyme EO (*Thymus vulgaris*); Lavender EO (*Lavandula angustifolia*)	In vitro In vivo Intramammary and External applications (oils mixed in vaseline)	*Staphylococcus* sp. *And Streptococcus* sp.	External use of these oils in vaseline resulted in a greater antibacterial action, for a 100% recovery rate with thymus essential oils.	Abboud et al. 2015 [51]
Cinnamon EO (*Cinnamomum zeylanicum*) Bergamot EO (*Citrus bergamia Risso*) Tasmanian blue gum EO (*Eucalyptus globulus*) Fennel EO (*Foeniculum vulgare*) Marjoram EO (*Origanum majorana*) Oregano EO (*Origanum vulgare*) Rosemary EO (*Rosmarinus officinalis*) Winter savory EO (*Satureja montana*) Thyme EO (*Thymus vulgaris*)	In vitro	*Staphylococcus aureus Staphylococcus chromogenes Staphylococcus sciuri Staphylococcus warneri Staphylococcus xylosus Escherichia coli*	The mixture containing *Thymus vulgaris* and *Winter savory* essential oils exhibited the best inhibitory activity against all the tested bacterial strains. The artificial mixtures composed of carvacrol/thymol, respectively carvacrol/thymol/p-cymene presented strong inhibition against *Staphylococcus aureus* and *Staphylococcus sciuri*	Fratini et al. 2014 [101]
Summer savory (*Satureja hortensis*) Silver fir (*Abies alba*)	In vitro	*Prototheca zopfii* isolates (from mastitic milk and bovine feces) *Prototheca wickerhami*	Fir oil is presented lower anti-algae activity as compared to summer savory	Bouari et al. 2011 [102]

**Table 2:** Summary of recent studies regarding the use of nanoparticles against bovine mastitis pathogens.

Type of Nanoparticles	Type of Study	Tested Pathogens	Effect	References
Silver nanoparticles (AgNPs)	in vitro	*Streptococcus agalactiae*	AgNPs showed reasonable antimicrobial and relatively low antibiofilm activities, while cinnamon oil showed high antimicrobial and antibiofilm against biofilms of *Streptococcus agalactiae* isolates.	Abd El-Aziz et al. 2021 [160]
Chitosan nanoparticles (Ch-NPs)	in vitro	*Staphylococcus aureus*	The smaller Ch-NPs were active in preventing *Staphylococcus aureus* from entering the cells, but they did not stimulate the formation of pro-inflammatory cytokines. The results support the assertion that Ch-NPs are an excellent bacteriostatic agent, capable of preventing the replication of bovine mastitis pathogens in the udder.	Orellano et al. 2021 [161]
Chitosan nanoparticles (Ch-NPs)	in vitro	*Pseudomonas* sp. strain isolated from bovine milk samples	The nanoparticles inhibited biofilm formation and could eliminate pre-existing mature biofilms.	Rivera Aguayo et al. 2020 [162]
Chitosan nanoparticles (Ch-NPs)	in vitro	*Staphylococcus aureus*	The antimicrobial activity of Ch-NP was higher than that of the native polymer used in the nanocomposites’ preparation. Ch-NPs impaired bacterial cell membranes and prevented the development of bacterial biofilms without impacting the viability of bovine cells.	Orellano et al, 2019 [163]
inc oxide nanoparticles (ZnO-NPs)	in vitro	*Staphylococcus aureus* *Escherichia coli Klebsiella pneumoniae* isolated from milk of affected cows.	At the same concentrations, capped dispersed ZnO-NPs demonstrated greater antibacterial activity against *Staphylococcus aureus, Escherichia coli Klebsiella pneumoniae* than non-capped nanoparticles. Gram-positive *Staphylococcus aureus* showed higher resistance to ZnO-NPs synthesized as compared to Gram-negative *Escherichia coli Klebsiella pneumoniae*.	Hozyen et al. 2019 [164]
Silver-nanoparticle-decorated quercetin nanoparticles (QA NPs)	in vitro	*Escherichia coli* multi-drug resistant strain isolated from a dairy cow with mastitis	QA NPs showed higher antibacterial and anti-biofilm properties in a multi-drug resistant *Escherichia coli* strain isolated from a dairy cow with mastitis, as compared to Ag NPs and quercetin alone.	Yu et al. 2018 [165]
Honey and Gold Nanoparticles	in vitro	*Methicillin-resistant (MRSA) and vancomycin-resistant (VRSA) coagulase-positive Staphylococcus aureus* isolated from contagious bovine clinical mastitis	AuNPs, 30 nm in size, presented visible anti- *Methicillin-resistant* (*MRSA*) and anti-*vancomycin-resistant* (*VRSA*) activities in vitro	Omara et al. 2017 [166]
